# Elevation of serum interleukin-1β levels as a potential indicator for malarial infection and severe malaria: a meta-analysis

**DOI:** 10.1186/s12936-022-04325-0

**Published:** 2022-10-29

**Authors:** Aongart Mahittikorn, Pattamaporn Kwankaew, Pongruj Rattaprasert, Kwuntida Uthaisar Kotepui, Frederick Ramirez Masangkay, Manas Kotepui

**Affiliations:** 1 Department of Protozoology, Faculty of Tropical Medicine, Mahidol University, Bangkok, Thailand; 2 Medical Technology, School of Allied Health Sciences, Walailak University, Tha Sala, Nakhon Si Thammarat, Thailand; 3 Department of Medical Technology, Faculty of Pharmacy, Royal and Pontifical University of Santo Tomas, Manila, Philippines

**Keywords:** IL-1β, Malaria severity, Severe malaria, Meta-analysis

## Abstract

**Background:**

Interleukin (IL)-1β is a proinflammatory cytokine that has a role in disease-related inflammation, including malaria. However, reports on the effect of IL-1β on malaria severity are inconsistent. Therefore, meta-analyses to compare differences in IL-1β levels between patients with severe malaria, patients with uncomplicated malaria and healthy controls were performed.

**Methods:**

The PRISMA standards were used to perform a systematic review and meta-analysis. A search of PubMed, Scopus, EMBASE and reference lists was conducted for articles providing data on IL-1β levels between patients with severe malaria, patients with uncomplicated malaria and healthy controls between January 1988 and March 2022, using a combination of search terms. The quality of all studies included in this review was determined using the Strengthening the Reporting of Observational Studies in Epidemiology statement: guidelines for reporting observational studies. The evidence was synthesized quantitatively and qualitatively. The differences in IL-1 levels across participant groups were recounted narratively for qualitative synthesis. For quantitative synthesis, the mean difference in IL-1β levels across groups of participants was calculated using a random effects meta-analysis. The publication bias was assessed using funnel plots, Egger’s test and a contour-enhanced funnel plot.

**Results:**

A total of 1281 articles were discovered, and the 17 that satisfied the inclusion criteria were included for syntheses. The meta-analysis results using data from 555 cases of severe malaria and 1059 cases of uncomplicated malaria showed that severe malaria had a higher mean of IL-1β levels than uncomplicated malaria (*P* < 0.01, pooled mean difference: 1.92 pg/mL, 95% confidence interval: 0.60–3.25 pg/mL, *I*^2^: 90.41%, 6 studies). The meta-analysis results using data from 542 cases of uncomplicated malaria and 455 healthy controls showed no difference in mean IL-1β levels between the two groups (*P* = 0.07, pooled mean difference: 1.42 pg/mL, 95% confidence interval: − 0.1–2.94 pg/mL, *I*^2^: 98.93%, 6 studies).

**Conclusion:**

The results from the meta-analysis revealed that IL-1β levels were higher in patients with severe malaria than in patients with uncomplicated malaria; however, IL-1β levels were similar in patients with uncomplicated malaria and healthy controls. Based on the limitations of the number of studies included in the meta-analysis and high levels of heterogeneity, further studies are needed to conclude that differences in IL-1β levels can be useful for monitoring the malaria severity.

**Supplementary Information:**

The online version contains supplementary material available at 10.1186/s12936-022-04325-0.

## Background

Severe malaria is defined as *Plasmodium falciparum* asexual parasitaemia in combination with one or more of the following complications: impaired consciousness, prostration, multiple convulsions, acidosis, hypoglycaemia, severe malarial anaemia, renal impairment, jaundice, pulmonary oedema, significant bleeding, shock or hyperparasitaemia [1]. Moreover, severe *Plasmodium vivax* and *Plasmodium knowlesi* malaria are classified similarly to falciparum malaria, except that parasite density criteria are not used [1].

Interleukin-1 (IL-1) is a critical regulator of inflammation because it is involved in a variety of innate immune responses [2]. According to the human sequencing algorithm technology, the IL-1 family includes 11 members: IL-1α, IL-1β, IL-1Ra, IL-18, IL-33, IL-36α, IL-36β, IL-36γ, IL-36Ra, IL-37 and IL-38, all of which have comparable or unique biological effects [3, 4]. There are two distinct forms of IL-1, IL-1α and IL-1β, which demonstrate similar biological functions [5]. Although IL-1α and IL-1β share only 27% amino acid sequence homology, they are physically similar and accomplish the same functions through the IL-1 type 1 receptor [6, 7]. IL-1β is primarily synthesized by macrophages, epithelial cells, fibroblasts and endothelial cells in response to pathogen-associated molecular patterns or damage-associated molecular patterns that signal through pattern recognition receptors (PRRs) [8]. IL-1β is expressed in a variety of tissues and cells, but is particularly abundant in macrophages and lymphoid organs, such as the bone marrow, thymus, spleen and lymph nodes. Additionally, it is secreted by non-lymphoid organs such as the digestive system, lung and liver [9, 10]. IL-1β is synthesized as a 269 amino acid precursor protein that is proteolytically cleaved by caspase-1 or other serine proteases activated during inflammation into the active form, which has 153 amino acids at the C-terminus [5, 11–15]. Significant effects of IL-1β include the following: (1) induction of endothelial cells; (2) activation of neutrophil diapedesis and (3) stimulation of cytokine production in the lymphocytes (T and B) [8].

IL-1β is a proinflammatory cytokine that has a role in disease-related inflammation, fever and discomfort [16, 17]. It participates in cellular processes such as proliferation, differentiation and death [18]. Additionally, IL-1β plays a key role in homeostasis, regulating appetite, sleep and body temperature [19]. High levels of IL-1β have been observed in patients with bacterial, viral, fungal and parasitic infections; several forms of malignancies; autoimmune disorders; trauma (surgery); ischaemic illnesses (myocardial infarction) and UV radiation [19]. Studies on IL-1β in the context of malaria are limited and the results are inconsistent; therefore, conclusions on IL-1β in various types of malaria are unclear. Therefore, meta-analyses to assess differences in IL-1β levels between various types of malaria, including between patients with severe malaria, patients with uncomplicated malaria and healthy controls were performed. The findings of this study will inform future research on IL-1β and its function in malaria infection and severity.

## Methods

### Protocol and search strategy

PRISMA standards were used to perform a systematic review and meta-analysis (Additional files 10, 11) [20]. The systematic review was registered at PROSPERO (CRD42022318871). A search of PubMed, Scopus, EMBASE and reference lists was conducted for articles providing data on IL-1β levels between patients with severe malaria, patients with uncomplicated malaria and healthy controls between January 1988 and March 2022. Broad search terms ‘(‘Interleukin 1 beta’ OR ‘Interleukin 1beta’ OR ‘IL-1 beta’ OR ‘Interleukin-1 beta’ OR Catabolin) AND (malaria OR plasmodium) were combined as a search strategy for different databases (Additional file 7: Table S1). Relevant article citations were manually searched to ensure that relevant articles were not missed. Additionally, authors of published articles were contacted to get data that could not be extracted directly from the source. The search began on 7 March 2022, and concluded on 20 March 2022.

### Eligibility criteria

To be considered for inclusion in the review, articles had to report IL-1β levels among patients with severe malaria, patients with uncomplicated malaria and healthy controls. The following articles were excluded: (i) studies providing data on IL-1β levels in patients with uncomplicated malaria only, (ii) studies providing data on IL-1β levels in pregnancy/cord blood, because these participants had a diverse immune response to malaria infection, (iii) in vitro studies measuring IL-1β production, (iv) studies from which IL-1β data could not be extracted, (v) conference abstracts on IL-1β, (vi) studies providing data on IL-1β levels in patients with asymptomatic malaria only, (vii) studies providing data on IL-1β levels in patients with severe malaria only and (viii) studies providing data on IL-1β levels after malaria treatment.

### Study selection and data extraction

The selection procedure began with the examination of titles and abstracts from three databases. The complete text of all qualified articles was then read and compared with the eligibility criteria. Additionally, the reference lists of papers included were evaluated to confirm that no study was omitted. Two authors (AM and MK) independently reviewed articles for inclusion and extracted data based on the following: first author name, publication year, research location, country, age range, number of patients, *Plasmodium* spp., IL-1β levels, technique used for diagnosing malaria and method used for quantifying IL-1β. Disagreements between the two authors were settled by consensus-building conversations.

### Critical appraisal

The quality of all studies included in this review was determined using the Strengthening the Reporting of Observational Studies in Epidemiology (STROBE) statement: guidelines for reporting observational studies [21]. Each study was evaluated on 22 items; a score of 1 (yes) or 0 (no) was awarded to each item and the aggregate of all values generated an overall quality score ranging from 0 to 22. The summed scores were classed as having high (> 75 percentile), moderate (50–75 percentile) or low (< 50 percentile) quality based on the total score.

### Data syntheses

The evidence was synthesized quantitatively and qualitatively. The differences in IL-1 levels across participant groups were recounted narratively for qualitative synthesis. For quantitative synthesis, the mean difference (MD) in IL-1β levels across groups of participants was calculated using a random effects meta-analysis, which is a type of meta-analysis where each study is weighted according to variations between and within studies. The mean and standard deviation (SD) of IL-1β levels were used to calculate MD in IL-1β levels between studies. When the median or interquartile range (IQR) was reported in the studies, the mean and SD were computed using the previously established approach [22]. If just SD was missing from the study, SD was derived from one or more studies with comparable mean values [23]. The degree of heterogeneity was determined using Cochran’s Q statistic and the *I*^2^ statistic. The forest plot was used to depict the MDs and confidence intervals (CIs). Outliers were detected using the leave-one-out strategy, which involved iteratively rerunning meta-analysis and deleting studies. The publication bias was assessed by visualizing funnel plot symmetry. The funnel plot would be asymmetric in case of publication bias [24, 25]. Egger’s test was used to assess funnel plot symmetry [25]. Egger’s test with statistical significance (*p* < 0.05) might indicate that funnel plot asymmetry was due to a small-study effect [26]. A contour-enhanced funnel plot was used to explore the cause (s) of funnel plot symmetry [27]. To investigate potential sources of variation, research designs, study sites, *Plasmodium* spp., age groups and methodologies for IL-1β measurement were used as covariates. Stata, version 17, was used to analyse the data (Stata Corporation, College Station, TX).

## Results

### Search results

**Fig. 1:**
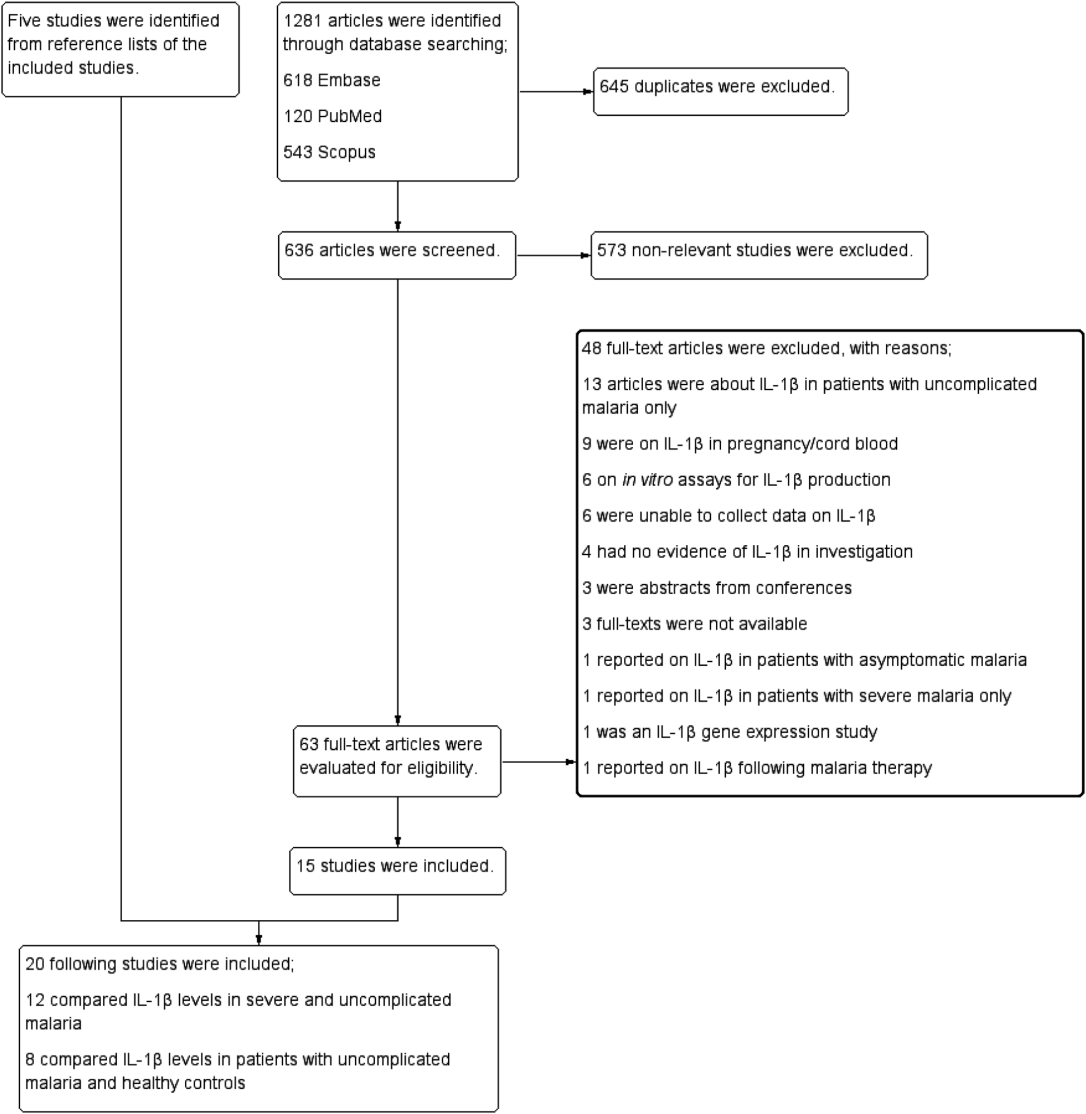
Study selection process

Through database searches, a total of 1281 articles were discovered, including 618 from Embase, 120 from PubMed and 543 from Scopus. After eliminating 645 duplicates, 636 articles were examined for titles and abstracts, followed by the exclusion of 573 irrelevant articles. The remaining 63 full-text articles were evaluated for eligibility, and 48 articles were eliminated for a variety of reasons; 13 articles were about IL-1β in patients with uncomplicated malaria only, 9 were on IL-1β in pregnancy/cord blood, 6 on in vitro assays for IL-1β production, 6 were unable to collect data on IL-1β, 4 had no evidence of IL-1β in the investigation, 3 were abstracts from conferences, 3 full-texts were not available, 1 reported on IL-1β in patients with asymptomatic malaria, 1 reported on IL-1β in patients with severe malaria only, 1 was an IL-1β gene expression study and 1 reported on IL-1β following malaria therapy. Fifteen studies [28–42] satisfied the inclusion criteria. Five articles [43–47] were found using the reference lists of the studies that were included. Finally, 20 studies [28–47] were included for qualitative and quantitative syntheses. These studies included 12 [28–36, 44, 46, 47] that compared IL-1β levels in severe and uncomplicated malaria and 8 [37–43, 45] that compared IL-1β levels in patients with uncomplicated malaria and healthy controls (Fig. 1).

### Characteristics and quality of the included studies

The characteristics of the included studies are shown in Table 1. Briefly, the included studies were published between 1994 and 2020. The included studies were case–control studies (8/20, 40%) [29, 33, 36, 37, 40, 43, 44, 46], cross-sectional studies (6/20, 30%) [30, 31, 34, 39, 41, 45], prospective observational studies (5/20, 25%) [28, 35, 38, 42, 47] and a cohort study [32]. The included studies were conducted in Africa (10/20, 50%) [28, 30, 31, 33, 34, 36, 38, 43, 44, 47], South America (4/20, 20%) [37, 40, 41, 45], Asia and Oceania [29, 35, 46], Europe [39, 42] and North America [32]. Most of the included studies enrolled patients with *P. falciparum* (13/20, 65%) [28, 30–36, 38, 39, 43, 44, 47] and children aged between less than 1 and 17 years (11/20, 55%) [28, 30–34, 36, 38, 44, 46, 47]. Finally, most of the included studies (16/20, 80%) used microscopy alone for to identify the malaria parasites [28–35, 37–40, 42–44, 47] and bead-based assay (13/20, 65%) for IL-1β quantification [28, 31–34, 38–41, 44–47]. Details of the included studies are shown in Additional file 8: Table S2. Among 20 studies included for the review, 19 were of high quality [28–42, 44–47] and 1 was of low quality [43] based on the STROBE checklist (Additional file 9: Table S3).

**Table 1:** Characteristics of the included studies

Characteristics	N	%
Study designs		
Case–control studies	8	40
Cross-sectional studies	6	30
Prospective observational studies	5	25
Cohort study	1	5
Study areas		
Africa	10	50
South America	4	20
Asia and Oceania	3	15
Europe	2	10
North America	1	5
*Plasmodium* spp.		
* P. falciparum*	13	65
* P. falciparum/P. vivax*	4	20
* P. vivax*	2	10
* P. knowlesi/P. falciparum*	1	5
Participants		
Children	11	55
Adults	7	35
All age groups	2	10
Methods for malaria detection		
Microscopy	16	80
Microscopy/PCR	4	20
Methods for TGF-β quantification		
Bead-based assay	13	65
ELISA	7	35

*ELISA* enzyme-linked immunosorbent assay, *PCR* polymerase chain reaction

### IL-1β levels in severe and uncomplicated malaria

**Fig. 2:**
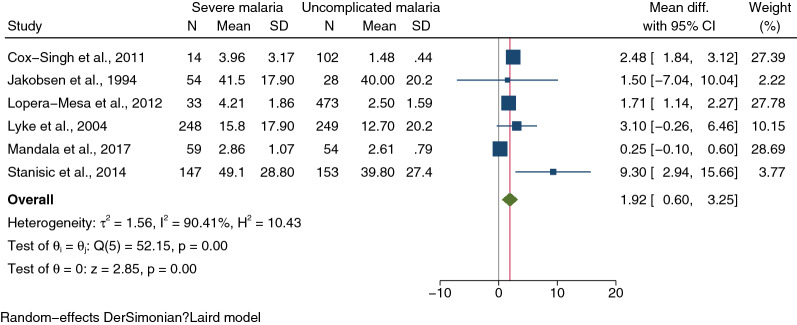
Forest plot demonstrating pooled MD of IL-1β levels (pg/mL) between patients with severe and uncomplicated malaria. Horizontal line (whiskers) encircling the square on both sides, CI; the squares’ centres and the values of the study’s effect sizes; green diamond, the overall effect size; the values of the overall effect size are indicated by the red vertical dashed line; vertical gray line with no effect on the overall composition (0); I^2^, H^2^, τb^2^, heterogeneity measures; under the green diamond**: **the test of I also known as the homogeneity test, is based on the Q statistic; the test of θ, the overall magnitude of the effect. *CI* confidence interval, *Mean Diff.* mean difference, *SD* standard deviation

Of the 20 studies included, 12 [28–36, 44, 46, 47] that compared IL-1β levels between severe and uncomplicated malaria were included for qualitative and quantitative syntheses. A qualitative synthesis of the 12 studies revealed that severe malaria had higher IL-1β levels than uncomplicated malaria [28, 29, 31, 32, 35, 46]. No difference between groups was demonstrated by other studies [30, 33, 34, 36, 44, 47]. Moreover, 6 studies [29, 30, 32, 33, 44, 46] provided quantitative data on IL-1β levels in severe (555 cases) and uncomplicated malaria (1059 cases) and were included in the meta-analysis. The meta-analysis results showed that severe malaria had a higher mean of IL-1β levels than uncomplicated malaria (*P* < 0.01, pooled MD: 1.92 pg/mL, 95% CI: 0.60–3.25 pg/mL, *I*^2^: 90.41%, 6 studies, Fig. 2).

**Fig. 3:**
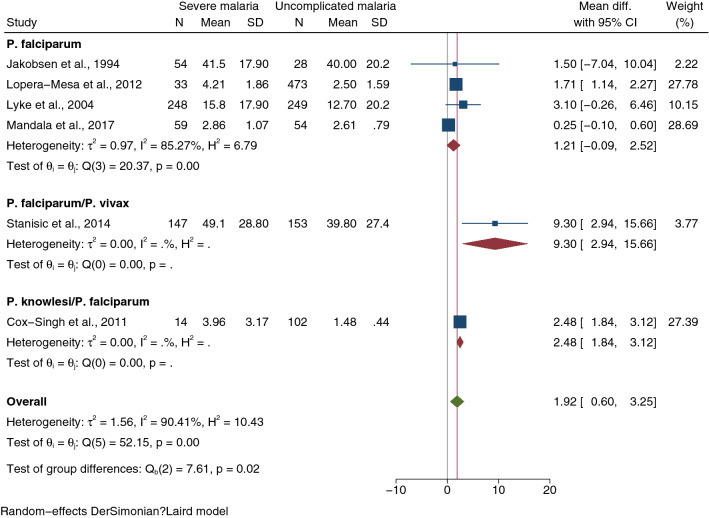
Forest plot demonstrating pooled MD of IL-1β levels (pg/mL) between patients with severe and uncomplicated malaria stratified by *Plasmodium* species. Horizontal line (whiskers) encircling the square on both sides, CI; the squares’ centres and the values of the study's effect sizes; green diamond, the overall effect size; the values of the overall effect size are indicated by the red vertical dashed line; vertical gray line with no effect on the overall composition (0); I^2^, H^2^, τb^2^, heterogeneity measures; under the crimson diamond**:** I^2^, H^2^, τb^2^, heterogeneity measures of subgroup; test of θi, the homogeneity test based on the Q statistic; under the green diamond**:** the test of θi also known as the homogeneity test, is based on the Q statistic; The test of θ, the overall magnitude of the effect. *CI* confidence interval, *Mean Diff.* mean difference, *SD* standard deviation

Meta-regression analysis using study design, continents, *Plasmodium* spp., age group and method for IL-1β quantification as covariates showed that only *Plasmodium* spp. confounded pooled MD (*P* = 0.047). Subgroup analysis of *Plasmodium* spp. showed no difference in IL-1β levels between the two groups in patients infected with *P. falciparum* (pooled MD: 1.21 pg/mL, 95% CI − 0.09–2.52 pg/mL, *I*^2^: 85.27%, four studies, Fig. 3). Data from other subgroups could not be interpreted because the number of studies was less than two.

### IL-1β levels in patients with uncomplicated malaria and healthy controls

**Fig. 4:**
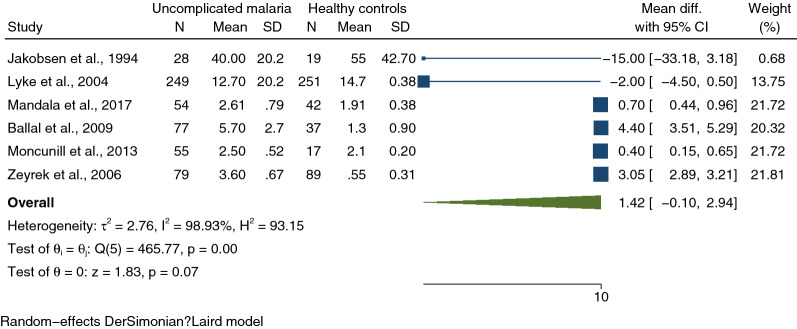
Forest plot demonstrating pooled MD of IL-1β levels (pg/mL) between uncomplicated and healthy control participants. The x-axis of the forest plot is on a logged scale 0–10, black x-axis line; the squares’ centres and the values of the study’s effect sizes in logged scale; green diamond; I^2^, H^2^, τb^2^, heterogeneity measures; under the green diamond: the test of I also known as the homogeneity test, is based on the Q statistic; the test of θ, the overall magnitude of the effect. *CI* confidence interval, *Mean Diff.* mean difference, *SD* standard deviation

Of 20 studies included in this study, 10 [30, 33, 37, 38, 40–45] that compared IL-1β levels between patients with uncomplicated malaria and healthy controls were included for qualitative and quantitative syntheses. A qualitative synthesis of 10 studies providing data on IL-1β levels between patients with uncomplicated malaria and healthy controls revealed that 6 [37, 41–45] reported higher IL-1β levels in patients with uncomplicated malaria than healthy controls. No difference in IL-1β levels between the two groups was reported by 4 studies [30, 33, 38, 40]. Of the 10 studies that compared IL-1β levels between patients with uncomplicated malaria and healthy controls, 6 [30, 33, 39, 42–44] provided quantitative data on IL-1β levels in uncomplicated malaria (542 cases) and healthy controls (455 individuals). Meta-analysis results showed no difference in mean IL-1β levels between the two groups (*P* = 0.07, pooled MD: 1.42 pg/mL, 95% CI − 0.1–2.94 pg/mL, *I*^2^: 98.93%, 6 studies, Fig. 4).

**Fig. 5:**
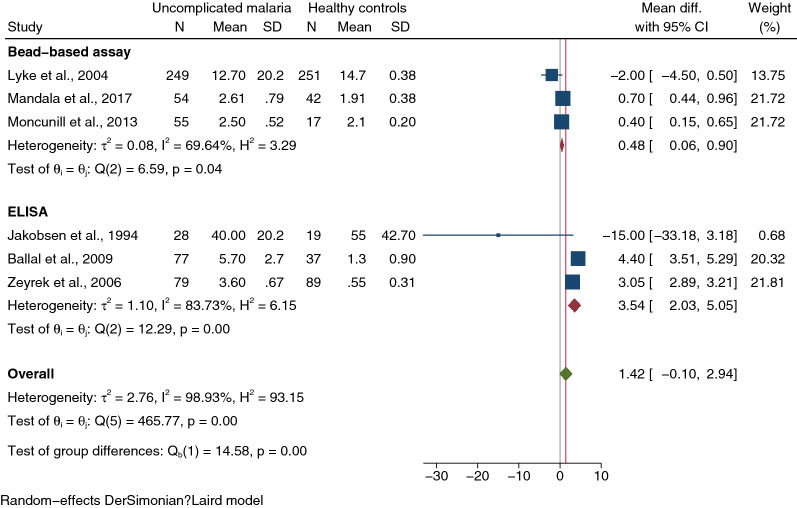
Forest plot demonstrating pooled MD of IL-1β levels (pg/mL) between patients with severe and uncomplicated malaria stratified by methods for IL-1β measurement. Horizontal line (whiskers) encircling the square on both sides, CI; the squares’ centres and the values of the study’s effect sizes; Green diamond, the overall effect size; the values of the overall effect size are indicated by the red vertical dashed line; vertical gray line with no effect on the overall composition (0); I^2^, H^2^, τb^2^, heterogeneity measures; under the crimson diamond**:** I^2^, H^2^, τb^2^, heterogeneity measures of subgroup; test of θi, the homogeneity test based on the Q statistic; under the green diamond**:** the test of θi also known as the homogeneity test, is based on the Q statistic; The test of θ, the overall magnitude of the effect. *CI* confidence interval, *Mean Diff.* mean difference, *SD* standard deviation

Meta-regression analysis using study design, continents, *Plasmodium* spp., age group and method for IL-1β quantification as covariates showed that only method for IL-1β quantification confounded pooled MD (*P* < 0.001). Subgroup analysis of the method for IL-1β quantification showed a subgroup difference (*P* < 0.01). A higher mean of IL-1β levels in uncomplicated malaria than healthy controls was observed in studies using the bead-based assay (pooled MD: 0.48 pg/mL, 95% CI 0.06–0.90 pg/mL, *I*^2^: 69.64%, three studies, Fig. 5) and studies using ELISA for IL-1β quantification (pooled MD: 3.54 pg/mL, 95% CI 2.03–5.05 pg/mL, *I*^2^: 83.73%, three studies, Fig. 5).

### Sensitivity analysis and certainty of the evidence

The sensitivity analysis using the leave-one-out method showed robust meta-analysis results, demonstrating a higher mean of IL-1β levels in severe malaria than uncomplicated malaria (*P* < 0.05 when each study was removed and the meta-analysis was rerun, 6 studies, Additional file 1: Fig. S1). However, meta-analysis results demonstrating differences in mean IL-1β levels between patients with uncomplicated malaria and healthy controls were not robust. *P* < 0.05 when three studies [30, 33, 42] were removed and the meta-analysis was rerun, *P* > 0.05 when three studies [39, 43, 44] were removed and the meta-analysis was rerun (Additional file 2: Fig. S2).

### Reporting bias

Reporting bias (publication bias) was assessed by visualizing funnel plot asymmetry, Egger’s test, Begg’s rank test and contour-enhanced funnel plot. For the meta-analysis of the difference in mean of IL-1β levels between severe malaria and uncomplicated malaria, the funnel plot was asymmetric (Additional file 3: Fig. S3); Egger’s test demonstrated no small-study effects (*P* = 0.07) and contour-enhanced funnel plot demonstrated the distribution of the MDs in both significant and non-significant areas (Additional file 4: Fig. S4), indicating that funnel plot asymmetry was due to other causes, such as heterogeneity of the MDs in the included studies, rather than publication bias.

For the meta-analysis of the difference in mean of IL-1β levels between patients with uncomplicated malaria and healthy controls, the funnel plot was asymmetric (Additional file 5: Fig. S5); Egger’s test demonstrated small-study effects (*P* = 0.03) and contour-enhanced funnel plot demonstrated the distribution of the MDs in both significant and non-significant areas (Additional file 6: Fig. S6), indicating that the cause of funnel plot asymmetry was due to publication bias and heterogeneity of the MDs in the included studies.

## Discussion

The meta-analysis confirmed that patients with severe malaria had higher IL-1β levels than those with uncomplicated malaria. Studies have indicated that IL-1β plays a crucial role in parasite clearance when combined with other cytokines, including interferon-gamma (IFN-γ), IL-2, IL-12 and TNF-α [48–50]. In the recent meta-analysis, significantly increased TNF-α [51] and decreased IL-12 levels were found in patients with severe malaria compared with patients with uncomplicated malaria. These results indicate that increased TNF-α and IL-1β production contributes to the pathogenesis of severe malaria. However, the overall meta-analysis result confirmed higher IL-1β levels than those in uncomplicated malaria. There were high levels of heterogeneity in IL-1β levels in the studies included in the meta-analysis (90.41%). The meta-regression analysis was used to test whether study design, continents, *Plasmodium* spp., age group and method for IL-1β quantification were confounders in the meta-analysis. Unfortunately, only *Plasmodium* spp. was a candidate confounder in the meta-analysis, and the results showed no difference in IL-1β levels between patients with severe and uncomplicated malaria caused by *P. falciparum* based on 4 studies [30, 32, 33, 44]. Because a limited number of studies were included in the meta-analysis, it could not be conclude that the infection by different *Plasmodium* spp. causes differences in IL-1β levels. There was a possibility that infection with different *Plasmodium* spp. may cause differences in IL-1β levels. For example, there was a low detection rate of IL-1β levels in patients infected with *P. knowlesi*, *P. vivax* and *P. falciparum*; however, the rate was higher for patients infected with *P. falciparum* than *P. vivax* and *P. knowlesi* [29]. Further studies are needed to confirm the differences in *Plasmodium* spp. and different cytokine levels in malaria severity.

In the literature, inconsistent reports exist on the influence of IL-1β on malaria severity. Studies have reported an increase in IL-1β in patients with severe malaria, notably cerebral malaria [31, 35]. In cerebral malaria, increased IL-1β production was shown to associate with malaria pathogenesis [52, 53]. Increased IL-1β levels in cerebral malaria were either directly or indirectly connected with brain oedema [54]. Vogetseder et al. reported that anti-malarial therapy for 5 d decreased IL-1β levels in patients with severe malaria, indicating that increased IL-1β levels involve malaria severity [55]. However, some studies indicated that no statistically significant differences in IL-1β levels existed between patients with different severity of malaria and healthy controls [28, 30]. Lyke et al. suggested that no correlation between IL-1β levels and malaria severity might be because IL-1β was downregulated by IL-10 [33].

Meta-analysis results confirmed that patients with uncomplicated malaria had comparable IL-1β levels to healthy controls. Nevertheless, a high level of heterogeneity in IL-1β levels was observed in the studies included in the meta-analysis (98.93%). The meta-regression analysis was used to test whether study design, continents, *Plasmodium* spp., age group and method for IL-1β quantification were confounder in the meta-analysis. We found that the method for IL-1β quantification was a confounder in the meta-analysis, and the results showed higher IL-1β levels in uncomplicated malaria than healthy controls in studies using both the bead-based assay and ELISA for IL-1β quantification. There was a difference in pooled MD in studies using the bead-based assay (0.48 pg/mL) and those using ELISA (3.54 pg/mL) for IL-1β quantification. The reason for the difference in the performance of the bead-based assay and ELISA for the detection of IL-1β is unknown. The bead-based assay is suitable for detection of several cytokines in a single platform, and ELISA can detect only one cytokine in a single platform [56]. Therefore, bead-based assays are preferred for screening several cytokines and may become increasingly commonplace [57].

The study has limitations. First, although changes in IL-1β levels in several groups of patients were examined, each cytokine performs a distinct function and contributes to a complex cytokine network. Therefore, predicting an association between cytokine levels and malaria severity is challenging. Second, the heterogeneity of outcome between the studies included in the meta-analysis may restrict the conclusion reached. The study was also limited due to publication bias, as indicated by the funnel plot. Third, publication bias was caused due to small-study effects in the meta-analysis of MDs between patients with uncomplicated malaria and healthy controls, indicating the need for more studies providing data on IL-1β levels in the meta-analysis. Fourth, the meta-analysis results revealing a higher mean of IL-1β levels in severe malaria compared with uncomplicated malaria had more certainty than those demonstrating a difference in mean IL-1β levels between patients with uncomplicated malaria and healthy controls. Fifth, the study excluded studies with pregnant women from the analysis. Future studies to determine differences in IL-1β levels in malaria in pregnancy are suggested, because pregnant women are a frequently neglected and highly vulnerable population.

## Conclusion

The meta-analysis results suggested that IL-1β levels were higher in patients with severe malaria than patients with uncomplicated malaria. However, IL-1β levels were not different between patients with uncomplicated malaria and healthy controls. Based on the limitations of the number of studies included in the meta-analysis and high levels of heterogeneity, further studies are needed to conclude that differences in IL-1β levels can be useful for monitoring malaria severity.

## Supplementary Information

**Additional file 1: Figure S1.** Sensitivity analysis using the leave-one-out method demonstrated the difference in mean IL-1β levels (pg/mL) between patients with severe malaria and those with uncomplicated malaria after excluding each study. Horizontal green line extending on either side of the green dot, CI; The green dots, the values of the overall effect size. CI: confidence interval; Mean Diff.: mean difference (MD); red vertical line: the overall effect size.

**Additional file 2: Figure S2.** Sensitivity analysis using the leave-one-out method demonstrated the difference in mean IL-1β levels (pg/mL) between patients with uncomplicated malaria and healthy control participants after excluding each study. Horizontal green line extending on either side of the green dot, CI; the green dots, the values of the overall effect size. CI: confidence interval; Mean Diff.: mean difference (MD); red vertical line, the overall effect size.

**Additional file 3: Figure S3.** Funnel plot of studies included in the meta-analysis of MD of IL-1β levels (pg/mL) between severe and uncomplicated malaria. CI: confidence interval; Mean Diff.: mean difference (MD); estimated θ_IV_: the overall effect size.

**Additional file 4: Figure S4.** Contour enhanced funnel plot of studies included in the meta-analysis of MD of IL-1β levels (pg/mL) between severe and uncomplicated malaria. CI: confidence interval; Mean Diff.: mean difference (MD).

**Additional file 5: Figure S5.** Funnel plot of studies included in the meta-analysis of MD of IL-1β levels (pg/mL) between uncomplicated malaria and healthy control participants. CI: confidence interval; Mean Diff.: mean difference (MD); estimated θ_IV_: the overall effect size.

**Additional file 6: Figure S6.** Contour enhanced funnel plot of studies included in the meta-analysis of MD of IL-1β levels (pg/mL) between uncomplicated malaria and healthy control participants. CI: confidence interval; Mean Diff.: mean difference (MD).

**Additional file 7: Table S1.** Search terms.

**Additional file 8: Table S2.** Quality of the included studies.

**Additional file 9: Table S3.** Details of the included studies.

**Additional file 10.** PRISMA 2020 abstract checklist.

**Additional file 11.** PRISMA 2020 checklist.

## Data Availability

All data and related materials are presented in this manuscript.

## Abbreviations

CIs: Confidence intervals
DAMPs: Damage-associated molecular patterns
IFN-γ: Interferon gamma
IL: Interleukin
IQR: Interquartile range
MD: Mean difference
PAMPs: Pathogen-associated molecular patterns
PRRs: Pattern recognition receptors
SD: Standard deviation
STROBE: Strengthening the Reporting of Observational Studies in Epidemiology
