# Supplementary figures and images for: Elevation of serum interleukin-1β levels as a potential indicator for malarial infection and severe malaria: a meta-analysis
Source: Malar J. 2022 Oct 29;21:308. doi: 10.1186/s12936-022-04325-0 (PMC9617441; doi:10.1186/s12936-022-04325-0)

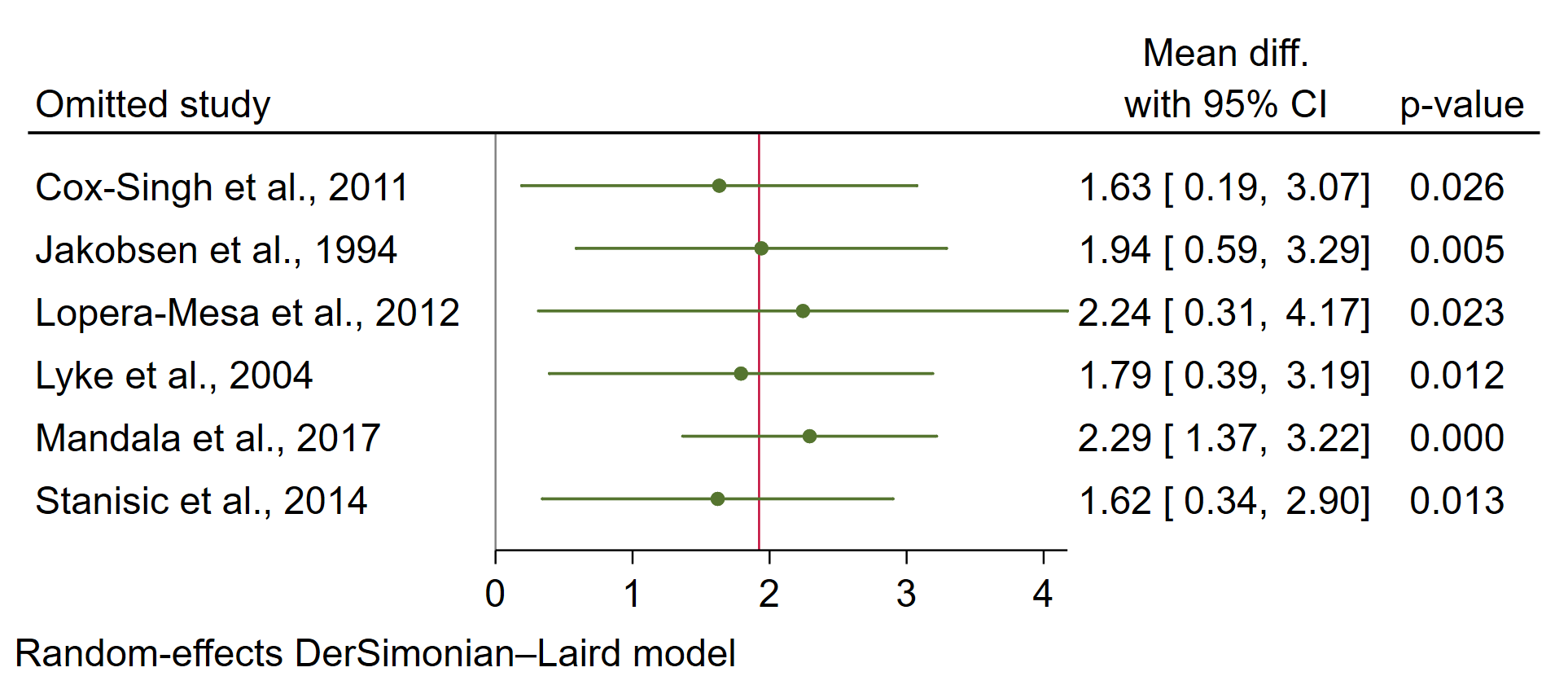

Supplement: Supplementary file 1 — Additional file 1: Figure S1. Sensitivity analysis using the leave-one-out method demonstrated the difference in mean IL-1β levels (pg/mL) between patients with severe malaria and those with uncomplicated malaria after excluding each study. Horizontal green line extending on either side of the green dot, CI; The green dots, the values of the overall effect size. CI: confidence interval; Mean Diff.: mean difference (MD); red vertical line: the overall effect size. [file 12936_2022_4325_MOESM1_ESM.tif]

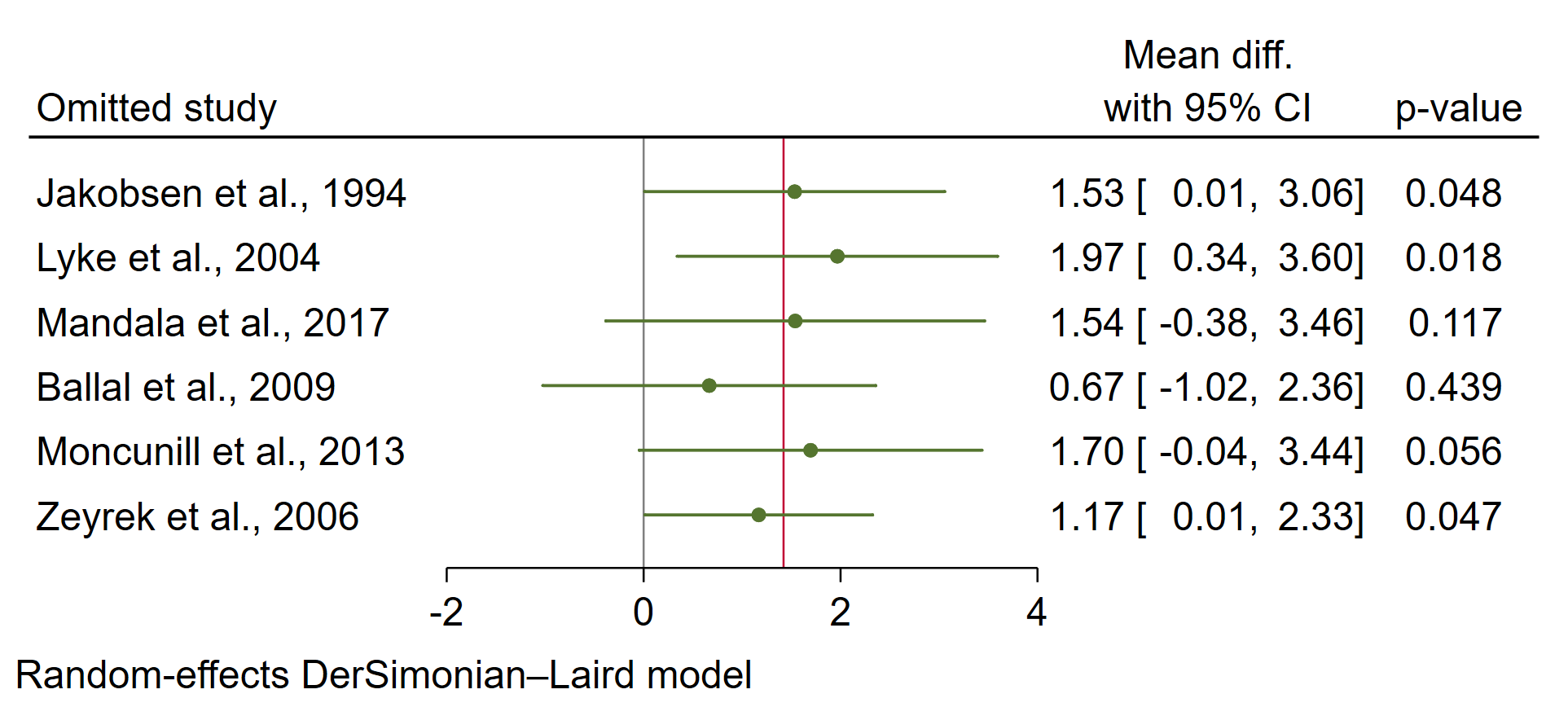

Supplement: Supplementary file 2 — Additional file 2: Figure S2. Sensitivity analysis using the leave-one-out method demonstrated the difference in mean IL-1β levels (pg/mL) between patients with uncomplicated malaria and healthy control participants after excluding each study. Horizontal green line extending on either side of the green dot, CI; the green dots, the values of the overall effect size. CI: confidence interval; Mean Diff.: mean difference (MD); red vertical line, the overall effect size. [file 12936_2022_4325_MOESM2_ESM.tif]

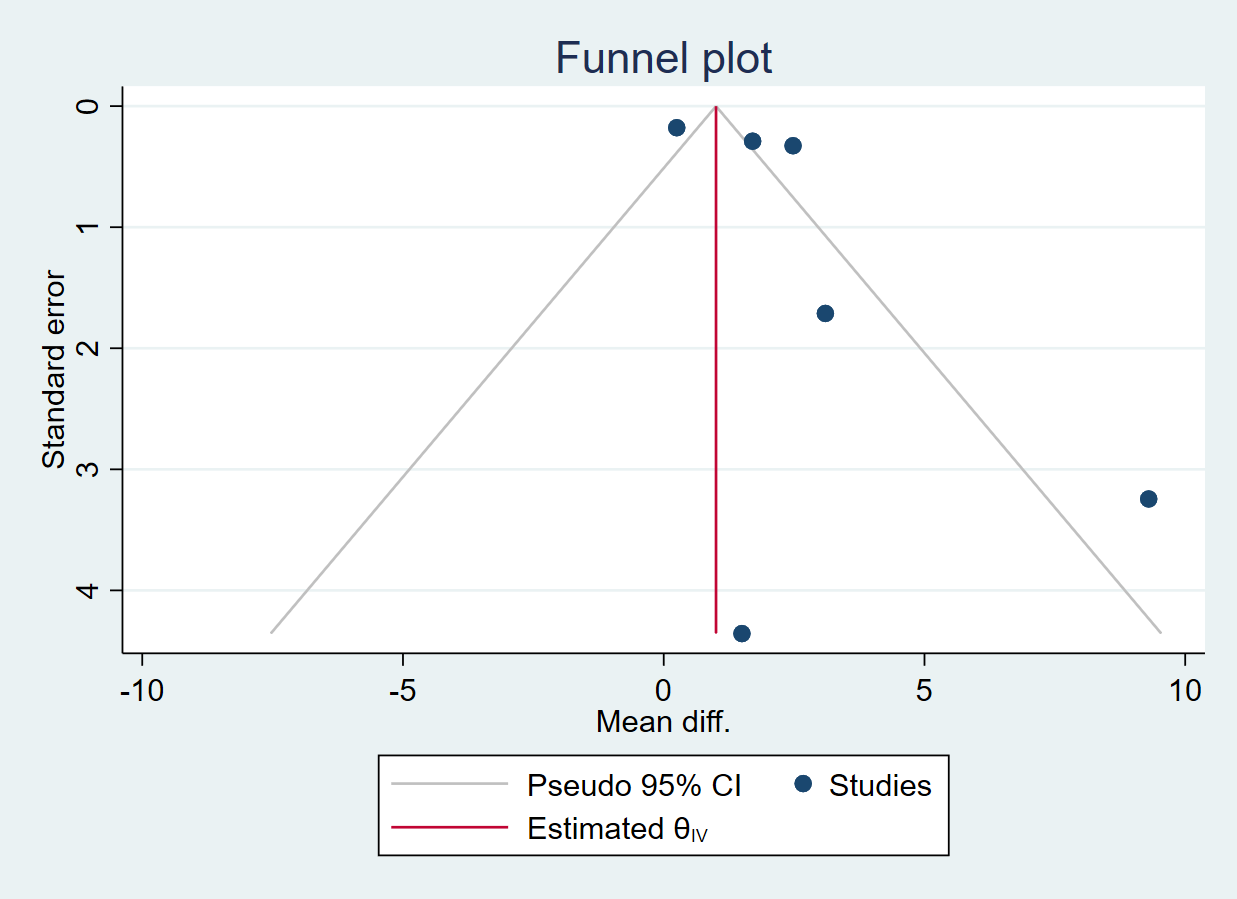

Supplement: Supplementary file 3 — Additional file 3: Figure S3. Funnel plot of studies included in the meta-analysis of MD of IL-1β levels (pg/mL) between severe and uncomplicated malaria. CI: confidence interval; Mean Diff.: mean difference (MD); estimated θIV: the overall effect size. [file 12936_2022_4325_MOESM3_ESM.tif]

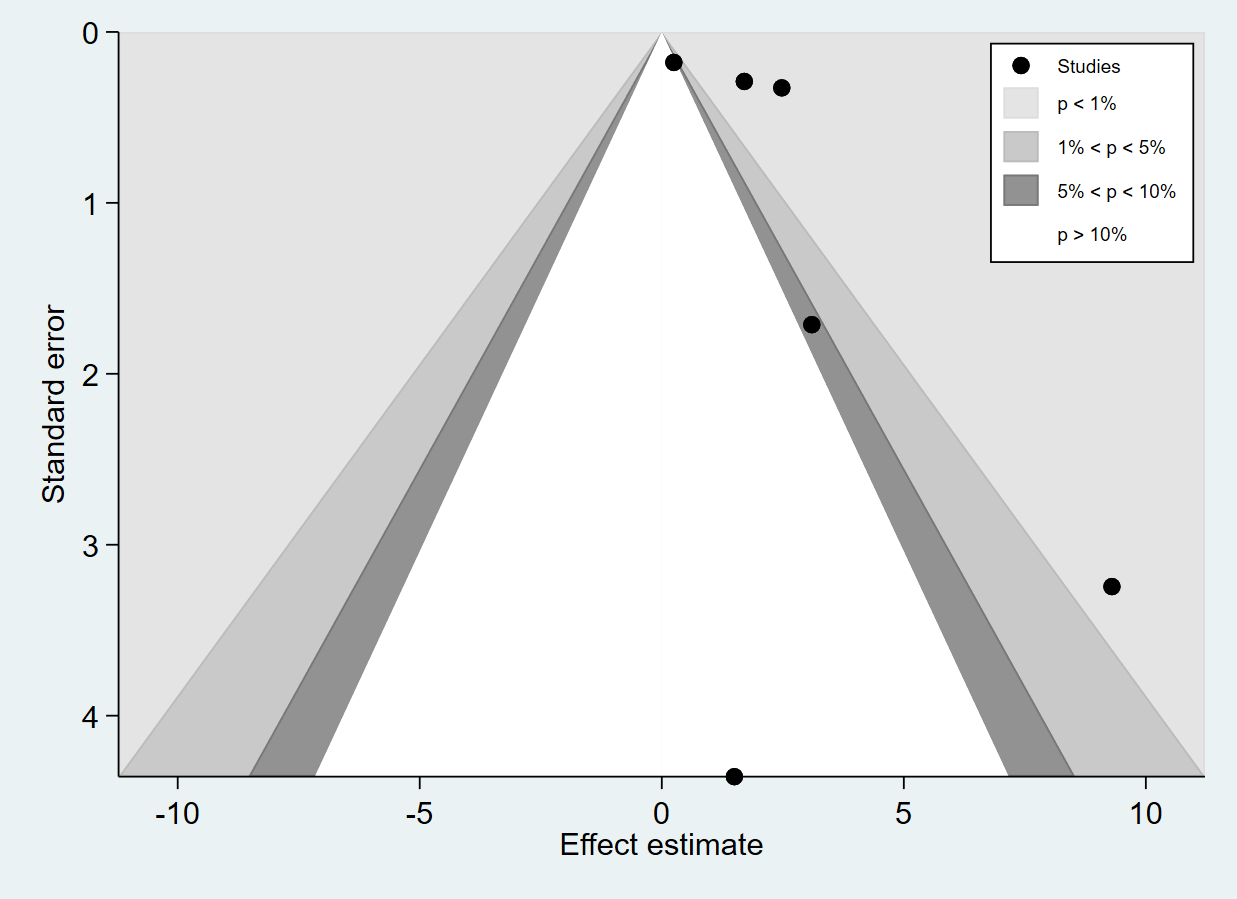

Supplement: Supplementary file 4 — Additional file 4: Figure S4. Contour enhanced funnel plot of studies included in the meta-analysis of MD of IL-1β levels (pg/mL) between severe and uncomplicated malaria. CI: confidence interval; Mean Diff.: mean difference (MD). [file 12936_2022_4325_MOESM4_ESM.tif]

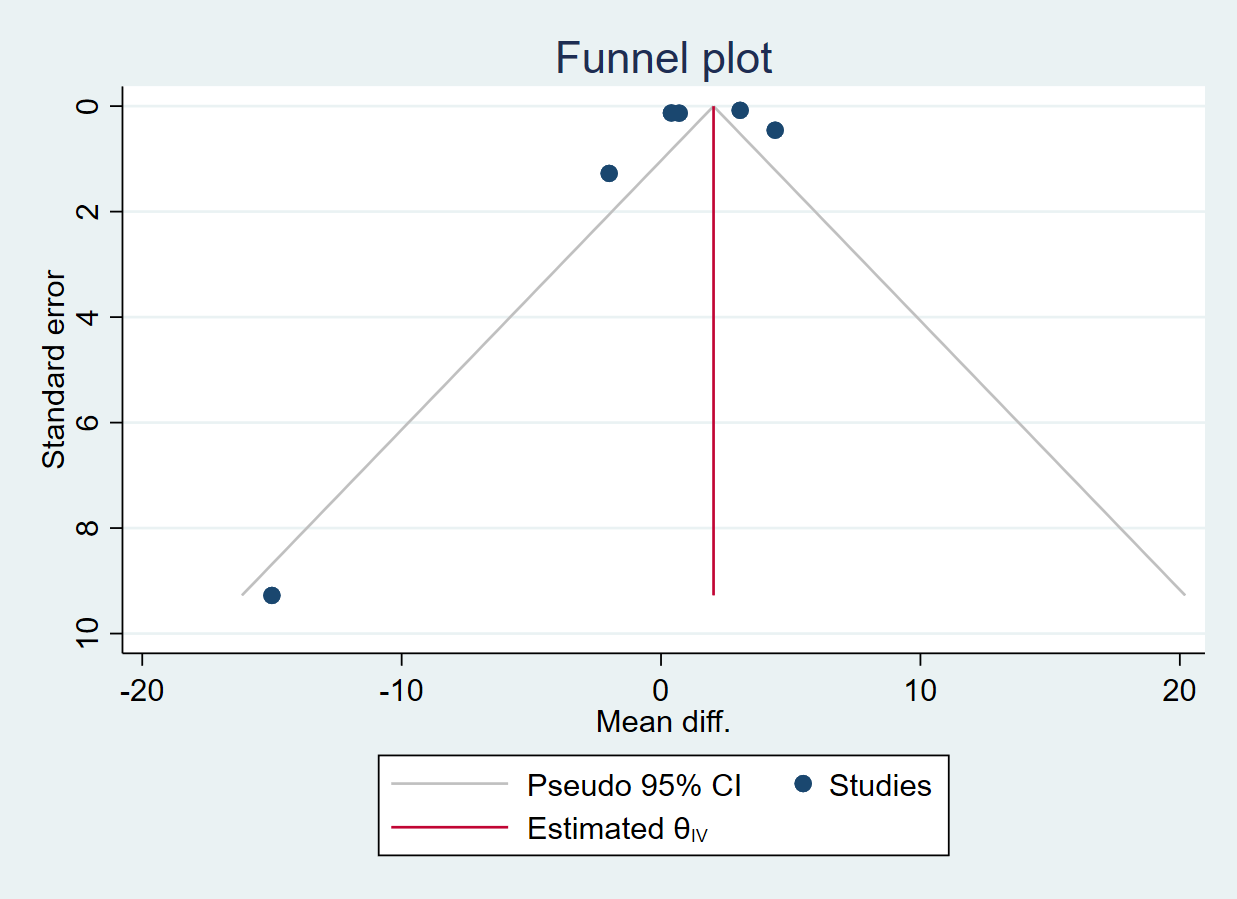

Supplement: Supplementary file 5 — Additional file 5: Figure S5. Funnel plot of studies included in the meta-analysis of MD of IL-1β levels (pg/mL) between uncomplicated malaria and healthy control participants. CI: confidence interval; Mean Diff.: mean difference (MD); estimated θIV: the overall effect size. [file 12936_2022_4325_MOESM5_ESM.tif]

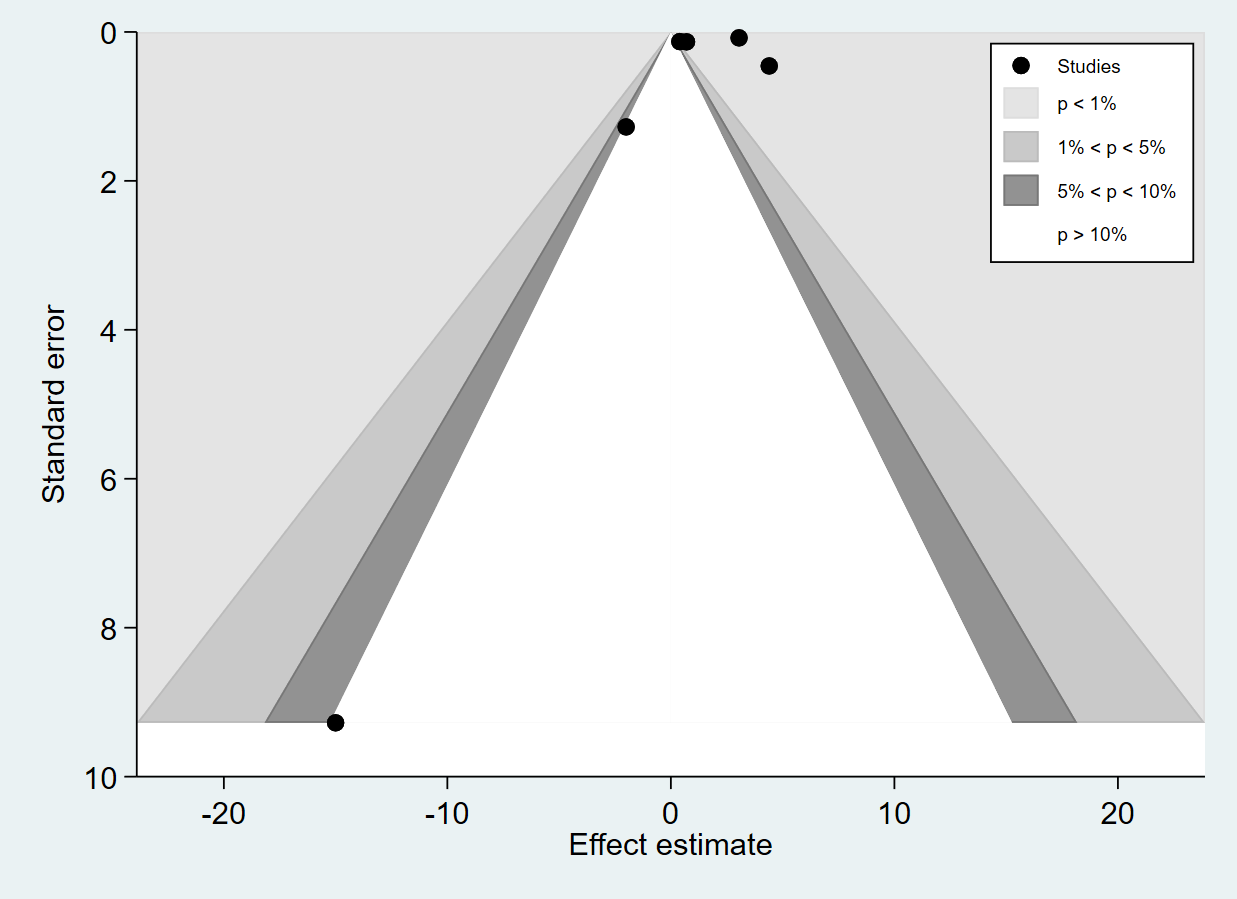

Supplement: Supplementary file 6 — Additional file 6: Figure S6. Contour enhanced funnel plot of studies included in the meta-analysis of MD of IL-1β levels (pg/mL) between uncomplicated malaria and healthy control participants. CI: confidence interval; Mean Diff.: mean difference (MD). [file 12936_2022_4325_MOESM6_ESM.tif]
