# Supplementary material for: Elevation of serum interleukin-1β levels as a potential indicator for malarial infection and severe malaria: a meta-analysis
Source: Malar J. 2022 Oct 29;21:308. doi: 10.1186/s12936-022-04325-0 (PMC9617441; doi:10.1186/s12936-022-04325-0)
Supplement: Supplementary file 7 — Additional file 7: Table S1. Search terms. [file 12936_2022_4325_MOESM7_ESM.docx]

**Elevation of serum interleukin-1β levels as a potential indicator for malarial infection and severe malaria: A meta-analysis**

Aongart Mahittikorn^1, †^, Pattamaporn Kwankaew^2, †^, Pongruj Rattaprasert^1^, Kwuntida Uthaisar Kotepui^2^, Frederick Ramirez Masangkay^3^, Manas Kotepui^2*^

^1^ Department of Protozoology, Faculty of Tropical Medicine, Mahidol University, Bangkok, Thailand

^2^ Medical Technology, School of Allied Health Sciences, Walailak University, Tha Sala, Nakhon Si Thammarat, Thailand

^3^ Department of Medical Technology, Faculty of Pharmacy, Royal and Pontifical University of Santo Tomas, Manila, Philippines

^*^Correspondence: manas.ko@wu.ac.th

AM: aongart.mah@mahidol.ac.th

PK: pattamaporn.kw@wu.ac.th

PR: pongruj.rat@mahidol.ac.th

KUK: kwuntida.ut@wu.ac.th

FRM: frederick_masangkay2002@yahoo.com

† These authors contributed equally to this work

**Table S1. Search term**

| **Databases** | **Search terms/Search strategy** | **Date** |
| --- | --- | --- |
| MEDLINE (via PubMed) | ("Interleukin 1 beta" OR "Interleukin 1beta" OR "IL-1 beta" OR "Interleukin-1 beta" OR "Interleukin 1 beta" OR Catabolin) AND (malaria OR plasmodium)  Search results: 120 | 7-20 March 2022 |
| Scopus | ("Interleukin 1 beta" OR "Interleukin 1beta" OR "IL-1 beta" OR "Interleukin-1 beta" OR "Interleukin 1 beta" OR Catabolin) AND (malaria OR plasmodium)  Search option: Title, abstract, keywords  Search results: 543 | 7-20 March 2022 |
| Embase | ("Interleukin 1 beta" OR "Interleukin 1beta" OR "IL-1 beta" OR "Interleukin-1 beta" OR "Interleukin 1 beta" OR Catabolin) AND (malaria OR plasmodium)  Search option: All fields  Search results: 618 | 7-20 March 2022 |
