# Supplementary material for: Elevation of serum interleukin-1β levels as a potential indicator for malarial infection and severe malaria: a meta-analysis
Source: Malar J. 2022 Oct 29;21:308. doi: 10.1186/s12936-022-04325-0 (PMC9617441; doi:10.1186/s12936-022-04325-0)
Supplement: Supplementary file 9 — Additional file 9: Table S3. Details of the included studies. [file 12936_2022_4325_MOESM9_ESM.docx]

**Elevation of serum interleukin-1β levels as a potential indicator for malarial infection and severe malaria: A meta-analysis**

Aongart Mahittikorn^1, †^, Pattamaporn Kwankaew^2, †^, Pongruj Rattaprasert^1^, Kwuntida Uthaisar Kotepui^2^, Frederick Ramirez Masangkay^3^, Manas Kotepui^2*^

^1^ Department of Protozoology, Faculty of Tropical Medicine, Mahidol University, Bangkok, Thailand

^2^ Medical Technology, School of Allied Health Sciences, Walailak University, Tha Sala, Nakhon Si Thammarat, Thailand

^3^ Department of Medical Technology, Faculty of Pharmacy, Royal and Pontifical University of Santo Tomas, Manila, Philippines

^*^Correspondence: manas.ko@wu.ac.th

AM: aongart.mah@mahidol.ac.th

PK: pattamaporn.kw@wu.ac.th

PR: pongruj.rat@mahidol.ac.th

KUK: kwuntida.ut@wu.ac.th

FRM: frederick_masangkay2002@yahoo.com

† These authors contributed equally to this work

**Table S3. Quality of the included studies**

**Case-control studies**

|  | **Study** | **Score (out of 22)** | **Score (percentage)** | **Quality** |
| --- | --- | --- | --- | --- |
| 1. | Ballal et al., 2009 | 15 | 68.2 | Moderate |
| 2. | Costa et al., 2020 | 20 | 91 | High |
| 3. | Cox-Singh et al., 2011 | 19 | 86 | High |
| 4. | Lyke et al., 2004 | 21 | 95 | High |
| 5. | Mandala et al., 2017 | 20 | 91 | High |
| 6. | Rodrigues-da-Silva et al., 2014 | 19 | 86 | High |
| 7. | Rovira-Vallbona et al., 2012 | 21 | 95 | High |
| 8. | Stanisic et al., 2014 | 20 | 91 | High |

**Cross-sectional studies**

|  | **Study** | **Score (out of 22)** | **Score (percentage)** | **Quality** |
| --- | --- | --- | --- | --- |
| 1. | Jakobsen et al., 1994 | 19 | 86 | High |
| 2. | John et al., 2006 | 20 | 91 | High |
| 3. | Moncunill et al., 2013 | 20 | 91 | High |
| 4. | Ong’echa et al., 2011 | 19 | 86 | High |
| 5. | Sánchez-Arcila et al., 2014 | 21 | 95 | High |
| 6. | Pinna et al., 2018 | 19 | 86 | High |

**Prospective observational and cohort studies**

|  | **Study** | **Score (out of 22)** | **Score (percentage)** | **Quality** |
| --- | --- | --- | --- | --- |
| 1. | Armah et al., 2007 | 19 | 86 | High |
| 2. | Davenport et al., 2016 | 19 | 86 | High |
| 3. | Lopera-Mesa et al., 2012 | 21 | 95 | High |
| 4. | Prakash et al., 2006 | 19 | 86 | High |
| 5. | Thuma et al., 2011 | 22 | 100 | High |
| 6. | Zeyrek et al., 2006 | 18 | 82 | High |

STROBE: Strengthening the Reporting of Observational Studies in Epidemiology
