# Supplementary material for: Elevation of serum interleukin-1β levels as a potential indicator for malarial infection and severe malaria: a meta-analysis
Source: Malar J. 2022 Oct 29;21:308. doi: 10.1186/s12936-022-04325-0 (PMC9617441; doi:10.1186/s12936-022-04325-0)
Supplement: Supplementary file 10 — Additional file 10. PRISMA 2020 abstract checklist. [file 12936_2022_4325_MOESM10_ESM.docx]

**Elevation of serum interleukin-1β levels as a potential indicator for malarial infection and severe malaria: A meta-analysis**

Aongart Mahittikorn^1, †^, Pattamaporn Kwankaew^2, †^, Pongruj Rattaprasert^1^, Kwuntida Uthaisar Kotepui^2^, Frederick Ramirez Masangkay^3^, Manas Kotepui^2*^

^1^ Department of Protozoology, Faculty of Tropical Medicine, Mahidol University, Bangkok, Thailand

^2^ Medical Technology, School of Allied Health Sciences, Walailak University, Tha Sala, Nakhon Si Thammarat, Thailand

^3^ Department of Medical Technology, Faculty of Pharmacy, Royal and Pontifical University of Santo Tomas, Manila, Philippines

^*^Correspondence: manas.ko@wu.ac.th

AM: aongart.mah@mahidol.ac.th

PK: pattamaporn.kw@wu.ac.th

PR: pongruj.rat@mahidol.ac.th

KUK: kwuntida.ut@wu.ac.th

FRM: frederick_masangkay2002@yahoo.com

† These authors contributed equally to this work

| **Section and Topic** | **Item #** | **Checklist item** | **Reported (Yes/No)** |
| --- | --- | --- | --- |
| **TITLE** | | |  |
| Title | 1 | Identify the report as a systematic review. | Yes |
| **BACKGROUND** | | |  |
| Objectives | 2 | Provide an explicit statement of the main objective(s) or question(s) the review addresses. | Yes |
| **METHODS** | | |  |
| Eligibility criteria | 3 | Specify the inclusion and exclusion criteria for the review. | Yes |
| Information sources | 4 | Specify the information sources (e.g. databases, registers) used to identify studies and the date when each was last searched. | Yes |
| Risk of bias | 5 | Specify the methods used to assess risk of bias in the included studies. | Yes |
| Synthesis of results | 6 | Specify the methods used to present and synthesise results. | Yes |
| **RESULTS** | | |  |
| Included studies | 7 | Give the total number of included studies and participants and summarise relevant characteristics of studies. | Yes |
| Synthesis of results | 8 | Present results for main outcomes, preferably indicating the number of included studies and participants for each. If meta-analysis was done, report the summary estimate and confidence/credible interval. If comparing groups, indicate the direction of the effect (i.e. which group is favoured). | Yes |
| **DISCUSSION** | | |  |
| Limitations of evidence | 9 | Provide a brief summary of the limitations of the evidence included in the review (e.g. study risk of bias, inconsistency and imprecision). | Yes |
| Interpretation | 10 | Provide a general interpretation of the results and important implications. | Yes |
| **OTHER** | | |  |
| Funding | 11 | Specify the primary source of funding for the review. | Provided in the main manuscript |
| Registration | 12 | Provide the register name and registration number. | PROSPERO (CRD42022318871) |

*From:*  Page MJ, McKenzie JE, Bossuyt PM, Boutron I, Hoffmann TC, Mulrow CD, et al. The PRISMA 2020 statement: an updated guideline for reporting systematic reviews. BMJ 2021;372:n71. doi: 10.1136/bmj.n71

For more information, visit: <http://www.prisma-statement.org/>
